# Synthetic data in generalizable, learning-based neuroimaging

**DOI:** 10.1162/imag_a_00337

**Published:** 2024-11-19

**Authors:** Karthik Gopinath, Andrew Hoopes, Daniel C. Alexander, Steven E. Arnold, Yael Balbastre, Benjamin Billot, Adrià Casamitjana, You Cheng, Russ Yue Zhi Chua, Brian L. Edlow, Bruce Fischl, Harshvardhan Gazula, Malte Hoffmann, C. Dirk Keene, Seunghoi Kim, W. Taylor Kimberly, Sonia Laguna, Kathleen E. Larson, Koen Van Leemput, Oula Puonti, Livia M. Rodrigues, Matthew S. Rosen, Henry F. J. Tregidgo, Divya Varadarajan, Sean I. Young, Adrian V. Dalca, Juan Eugenio Iglesias

**Affiliations:** Massachusetts General Hospital, Harvard Medical School, Boston, MA, United States; Massachusetts Institute of Technology, Cambridge, MA, United States; University College London, London, England; Computer Science & Artificial Intelligence Laboratory, Massachusetts Institute of Technology, Cambridge, MA, United States; Universitat de Girona, Girona, Spain; University of Washington, Seattle, WA, United States; ETH Zürich, Zürich, Switzerland; Copenhagen University Hospital, København, Denmark; Universidade Estadual de Campinas, São Paulo, Brazil

**Keywords:** SynthSeg, SynthStrip, SynthMorph, EasyReg, SynthSR

## Abstract

Synthetic data have emerged as an attractive option for developing machine-learning methods in human neuroimaging, particularly in magnetic resonance imaging (MRI)—a modality where image contrast depends enormously on acquisition hardware and parameters. This retrospective paper reviews a family of recently proposed methods, based on synthetic data, for generalizable machine learning in brain MRI analysis. Central to this framework is the concept of domain randomization, which involves training neural networks on a vastly diverse array of synthetically generated images with random contrast properties. This technique has enabled robust, adaptable models that are capable of handling diverse MRI contrasts, resolutions, and pathologies, while working out-of-the-box, without retraining. We have successfully applied this method to tasks such as whole-brain segmentation (SynthSeg), skull-stripping (SynthStrip), registration (SynthMorph, EasyReg), super-resolution, and MR contrast transfer (SynthSR). Beyond these applications, the paper discusses other possible use cases and future work in our methodology. Neural networks trained with synthetic data enable the analysis of clinical MRI, including large retrospective datasets, while greatly alleviating (and sometimes eliminating) the need for substantial labeled datasets, and offer enormous potential as robust tools to address various research goals.

## 1 Introduction

As MRI provided researchers with the opportunity to study the human brain *in vivo*, open-source neuroimaging software emerged to enable quantitative analysis of imaging data at scale. Packages like FreeSurfer (Fischl, 2012), FSL (S. M. Smith et al., 2004), SPM (Ashburner & Friston, 2005), or AFNI (Cox, 1996) have facilitated large-scale studies of healthy aging, dementia, and neurological disorders (Dima et al., 2022; Frangou et al., 2022; The Alzheimer’s Disease Neuroimaging Initiative et al., 2015; Thompson et al., 2020). Other tools analyze scans of subjects with diseases that more severely alter the structure of the brain, such as strokes or tumors (Gordillo et al., 2013; Kamnitsas et al., 2017; Pereira et al., 2016). These tools have also increased the reproducibility of research, particularly when combined with public datasets, such as ADNI (Jack Jr. et al., 2008), HCP (Van Essen et al., 2013), the UK BioBank (Alfaro-Almagro et al., 2018), or BraTS (Menze et al., 2015).

Over the past decade, rapid advances in deep learning have transformed neuroimaging methods, in areas such as MRI reconstruction (Zhu et al., 2018), segmentation (Kamnitsas et al., 2017; Milletari et al., 2016), or registration (Balakrishnan et al., 2019; De Vos et al., 2019). Powered by convolutional neural networks (CNNs; LeCun et al., 1998) or, more recently, vision transformers (Z. Liu et al., 2021), these methods frequently achieve higher levels of performance than their classical counterparts. In addition, deep learning methods often have considerably shorter inference run times compared to classical methods, especially when running on graphics processing units (GPUs). This increased speed enables application at a larger scale or in time-sensitive applications, such as clinical fetal imaging (Ebner et al., 2020; Hoffmann, Abaci Turk, et al., 2021).

However, a major roadblock to the wider adoption of deep learning methods in neuroimaging is their sensitivity to the so-called “domain shift”—the drop in performance when models trained on one dataset are applied to other datasets with even slightly different image intensity profiles (Billot, Greve, Puonti, et al., 2023). The domain shift is particularly problematic in uncalibrated modalities like MRI, as opposed to computerized tomography, where voxel intensities correspond to physical Hounsfield units. In MRI, variations in image intensities arise from differences in hardware, pulse sequences, slice direction, or resolution.

While data augmentation (Shorten & Khoshgoftaar, 2019; A. Zhao et al., 2019) and domain adaptation techniques (Wang & Deng, 2018) mitigate the problem for smaller domain shifts, they do not close the domain gap—especially in more dramatic shifts. For this reason, most algorithms in neuroimaging packages (FreeSurfer, FSL, SPM) still rely on Bayesian methods that are robust against changes to MRI contrast (Ashburner & Friston, 2005; Fischl et al., 2002; Patenaude et al., 2011) or pathology (Agn et al., 2019; Van Leemput et al., 2001).

Recently, our group has proposed a solution to this problem based on “domain randomization” (Tobin et al., 2017; Tremblay et al., 2018). These methods rely on training neural networks with an extremely wide distribution of synthetic data simulated with random parameters, in our case, MRI contrast, resolution, and artifacts like bias field or motion. By randomizing these parameters at each iteration during training, the neural network learns *not* to expect a specific MRI contrast or resolution, thus becoming agnostic to them. With this strategy, real images appear to the trained neural network as just another variation of the wide distribution.

Domain randomization has the advantage that the trained neural networks are available to process new images “out of the box,” without retraining or domain adaptation. This feature makes domain randomization particularly appealing when publicly distributing software. In FreeSurfer, we currently distribute multiple domain randomization powered methods for an array of brain MRI analysis tasks, including segmentation (Billot, Greve, Puonti, et al., 2023; Billot, Magdamo, et al., 2023; Hoopes, Mora, et al., 2022; Kelley et al., 2024), registration (Hoffmann et al., 2022, 2023, 2024; Iglesias, 2023), super-resolution (Iglesias et al., 2020, 2022), and contrast transfer (Iglesias et al., 2023).

Throughout the rest of this article, we do not make any assumption on the specific architectures used for learning. Instead, we focus on the *data* that are used for training them, since architecture and training data are generally independent of each other. Of course, some architectures are more suitable to certain problems than other; the reader is referred to Alzubaidi et al. (2021), Khan et al. (2023), and Taye (2023) for a comprehensive review of this topic.

## 2 Background

Machine-learning models developed with partially representative datasets or rigid simulation environments often struggle to adapt to the unpredictability and complexity of real-world inputs. Consequently, integrating data synthesis into model training pipelines has become a wide-spread strategy to improve model generalization under such circumstances. In this section, we review existing efforts to generate training data in the context of both general computer vision and medical image analysis.

### 2.1 Synthetic data in computer vision

Many real-world imaging datasets lack the diversity and scope necessary to train universally robust solutions for computer vision tasks. This is in part because acquiring images with useful annotations—such as semantic segmentations, point-wise labels, or classifications—requires substantial manual effort and, in some cases, costly budgets. Consequently, several groups have developed large-scale, specialized datasets comprising training images and annotations generated through automated means.

Some of these datasets derive from spatial augmentation or corruption pipelines, for instance, producing image-annotation pairs for object anomaly and defect analysis (C.-L. Li et al., 2021). Alternatively, recent generative approaches learn to synthesize a distribution of high-quality images, in some cases containing features described by a input language prompt. This facilitates building specialized datasets for object detection entirely from scratch, manipulating images, or approximating missing data in existing cohorts (Azizi et al., 2023; Kar et al., 2019; G. Zhang et al., 2021).

Other synthetic data contributions use techniques in computer graphics to simulate images from 3D object representations. These methods compute ground-truth attributes, like pixel class, depth, and location, used to train models for autonomous vehicles (Alhaija et al., 2017; Das et al., 2023; Kundu et al., 2018; Ros et al., 2016; Sadeghi & Levine, 2016; Wu et al., 2017), human pose estimation (Hewitt et al., 2023; Varol et al., 2017), or facial analysis (Bae et al., 2023; Wood et al., 2021, 2022). Some of these approaches involve highly complex simulations. For instance, robotics models use physics engines to synthesize multimodal inputs from interactive environments with contact dynamics and constraints (Shah et al., 2018; Todorov et al., 2012). Similarly, synthetic scene generation creates customizable environments for the development and evaluation of autonomous driving models in controlled scenarios (Dosovitskiy et al., 2017). These techniques not only improve model robustness for realistic inference-time objectives, but also provide control over certain data biases and anonymity, especially for privacy-sensitive domains like healthcare or facial image analysis.

### 2.2 Applications of synthetic data in medical image analysis

Synthesis methods are used to overcome similar data scarcity challenges in medical imaging. These approaches predominantly use synthetic and augmented datasets of diverse human anatomies to balance imaging features or disease classes, reduce manual annotation efforts, and ultimately enhance model robustness for tasks such as segmentation, registration, and classification. Some applications develop structural datasets for anatomies, such as the heart (Al Khalil et al., 2020; Azizmohammadi et al., 2022; Xanthis et al., 2021), kidney (Brumer et al., 2022), vertebrae (Sun et al., 2023), and brain (Billot, Greve, Puonti, et al., 2023; Dorjsembe et al., 2024; Kossen et al., 2022), or underrepresented populations, such as pediatric data (de Dumast et al., 2022). Synthesis techniques are particularly valuable for obtaining data that are difficult to collect in clinical or research settings. For instance, numerous published datasets with generated lesions, and often annotations, include those that synthesize lung disease and COVID-19 lesions (Ghorbani et al., 2019; Y. Jiang et al., 2020; X. Li et al., 2009; Thambawita et al., 2022; Waheed et al., 2020; Zunair & Hamza, 2021), brain tumors and neurodegenerative conditions (Ahmad et al., 2022; Bernal et al., 2021; Prados et al., 2016; Salem et al., 2019; H.-C. Shin et al., 2018), breast cancer (Badano et al., 2018; Cha et al., 2020; Pezeshk et al., 2015; Sarno et al., 2021; Sauer et al., 2023; Sizikova et al., 2024), colon polyps (Thambawita et al., 2022), and liver lesions (Ben-Cohen et al., 2019; Frid-Adar et al., 2018).

Some applications of medical image synthesis employ style transfer techniques to alter the modality or contrast properties of real scans (J. Jiang et al., 2018; J. Li et al., 2022). For instance, CT images predicted from MRI scans are useful for radiotherapy planning (Boulanger et al., 2021), PET images generated from CT scans enhance liver lesion segmentation (Ben-Cohen et al., 2019), and generated X-rays can be used to reduce radiation dose in cardiac interventions (Azizmohammadi et al., 2022). Other methods aim to harmonize images from different scanners by converting them to a unified style or developing domain-agnostic models to maintain good performance despite domain shifts—thus facilitating subsequent harmonization or even rendering it unnecessary (C. Ma et al., 2019; Yamashita et al., 2021).

Similarly, synthetic corruption and downsampling of real data is useful in applications like image quality transfer (IQT), which estimates high-quality data from low-quality images, in a deterministic or probabilistic fashion. While IQT with paired low- and high-quality images acquired separately (e.g., on different devices) is possible, IQT based on synthetic downsampling is far more common as it only requires the high-quality scans (and also bypasses the need to accurately co-register the image pairs). IQT enables the automatic improvement of images acquired with portable or older MRI scanners with lower field strength (Alexander et al., 2017; Blumberg et al., 2018; Jones et al., 2018; Kim & Alexander, 2021; C.-H. Lin et al., 2018; Tanno et al., 2020). Several works have shown that combining real and synthetic scans endows IQT (both in its deterministic and probabilistic version) with increased generalization ability at test time (Figini et al., 2020; Kim et al., 2023; H. Lin et al., 2023).

### 2.3 Methods for synthesizing data in medical image analysis

Model-based techniques, one category of medical image synthesis, rely on hand-crafted pipelines to generate data based on known physical properties of the desired image distributions. The simplest of these approaches uses geometric and intensity augmentations of existing images or label maps (Shorten & Khoshgoftaar, 2019). Geometric augmentation samples a spatial transform that deforms anatomy, while intensity augmentation randomly varies the image intensity profile—for example, by randomly modifying the brightness, contrast, and gamma correction of the images; or by filtering them using random convolutional kernels (Xu et al., 2020) or shallow networks with random weights (Ouyang et al., 2022). More complex techniques leverage biophysical models of the imaging signal to simulate realistic acquisitions or use deformable image registration to induce longitudinal growth or atrophy within an existing scan (Graff, 2016; Jog et al., 2019; Kainz et al., 2019; Karaçali & Davatzikos, 2006; Khanal et al., 2017; Larson & Oguz, 2022; Sengupta et al., 2021; A. D. C. Smith et al., 2003; Teixeira et al., 2018).

Alternatively, recent generative deep-learning methods that learn to replicate spatial patterns in a training distribution are used to produce synthetic images that resemble real-world data. Even when actual data exist, these models can help in scenarios where data sharing is restricted due to privacy concerns, as synthetic images can often be disseminated with fewer limitations (Pinaya et al., 2022). Deep generative model architectures like generative adversarial networks (GANs) (Bowles et al., 2018; Y. Chen et al., 2022), variational autoencoders (VAEs) (Casale et al., 2018; Kingma & Welling, 2013), normalizing flows (Wilms et al., 2022), and diffusion probabilistic models can achieve high-resolution image synthesis (Chung et al., 2022; Ho et al., 2020; Kazerouni et al., 2023; Rombach et al., 2022). Conditional generative models can also be used to produce or augment images based on a prompt. For instance, given a label map, BrainSpade (Fernandez et al., 2022) generates brain scans conditioned on features of reference scan to control generated image style or the presence of certain pathology. RoentGen (Chambon et al., 2022) synthesizes chest X-rays with features based on an input natural language description.

A related approach to fully generative modeling is semi-supervised learning, where the same label maps for generative modeling are used to facilitate learning from numerous unlabeled examples and a small number of labeled ones. Semi-supervised learning approaches are particularly useful if the generative forward model is complex or otherwise challenging to simulate, leading to a large gap between synthesized and real data. Approaches that employ label maps for semi-supervision include supervision by denoising (SUD) (Young et al., 2023) and denoising diffusion probabilistic model (DDPM) variants (Chan et al., 2023) with applications to whole brain segmentation and cortical parcellation.

We emphasize, however, that the capacity of generative methods to synthesize diverse images is ultimately confined by the scope of data used to train it. As a result, the robustness of downstream models trained on generated outputs is constrained not only by the overall accuracy of the generator, but also by potential domain gaps that exist in real data.

## 3 Brain Image Synthesis Using Domain Randomization

To address training domain gaps in learning-based brain imaging methods, our group has applied a model-based approach for constructing entirely synthetic training datasets that feature anatomies, intensity distributions, and artifacts that extend well beyond the realistic range of medical images. This strategy facilitates training models capable of generalizing across a variety of *real* brain images.

### 3.1 Method

**Fig. 1.**
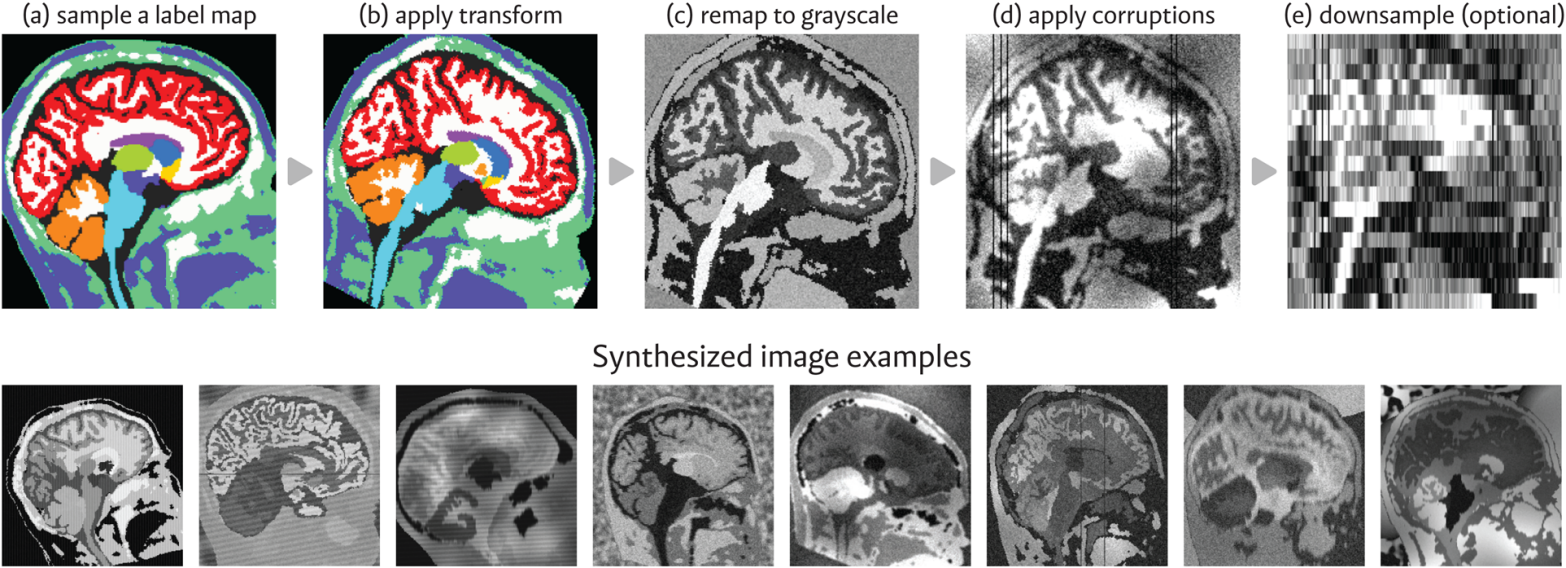
Top: Overview of our image synthesis process. First, we (a) sample a prior-generated whole-brain label map and (b) transform it with random affine and deformable spatial augmentations. Then, we (c) remap the labels to grayscale, drawing from random Gaussian distributions, and (d) simulate artifacts, such as noise, intensity bias, and smoothing. In some applications, we (e) resample the image to a new voxel spacing. Bottom: Series of images generated with random synthesis parameters, each derived from the *same* initial label map.

This framework requires a pre-computed set of whole-brain, anatomical label maps S, derived manually or automatically. To generate an image, we sample a label map s∼S and apply a domain randomization model following Figure 1. First, we generate a transformed map $s_{\phi }$ with varied anatomical morphology by applying to s a random affine and non-linear deformation pair ϕ. Next, we consider a model of tissue contrast inspired by the classical Bayesian segmentation literature (Ashburner & Friston, 2005) and generate an intensity distribution for each label in $s_{\phi }$, using uniformly sampled Gaussian parameters. With this random mixture model, we compute a grayscale image x by recoding voxel labels in $s_{\phi }$ with values sampled from their corresponding label-specific intensity distribution. Lastly, we augment x with randomly simulated image artifacts such as spatial blurring, added noise, bias field exponentiation, and slice corruption. In some cases, we manipulate the acquisition geometry, for instance by randomly cropping the field-of-view or resampling to a new voxel spacing in a sampled slice direction (axial, coronal, or sagittal).

We follow the standard deep learning setup, but instead of using acquired brain data, we use the synthetic images during training. As usual, we propagate the prediction error using ground truth annotations or an unsupervised loss function. For example, in some applications, components of the deformed label map $s_{\phi }$ can be used as targets to train a segmentation model. Alternatively, ϕ can be applied to original acquisitions to compute targets for an image reconstruction model.

### 3.2 Downstream model robustness

Brain image analysis models trained using this approach are broadly applicable and generalize well across MRI sequences and contrasts, field strengths, and scanner manufacturers—all without using real acquisitions during training or having to retrain networks for different domains. This generalizability enhances the utility of neuroimaging models as robust, deployable tools that clinicians and researchers alike can readily apply to their own data, without requiring specialized hardware or machine-learning expertise to fine-tune inflexible models for particular acquisition specifics.

Additionally, compared to supervised models developed with real images, those optimized with synthetic data and training targets derived from $s_{\phi }$ are less vulnerable to imperfections in ground-truth data, due to the inherent alignment between label maps and synthesized images. While not entirely resilient to major segmentation errors, this method mitigates common issues using conventional data, where small discrepancies between target labels and underlying image features often disrupt the learning process. Consequently, it minimizes the need to produce highly precise, manual segmentations for training.

We emphasize that while our approach improves model robustness across acquisition specifics, it may not completely bridge the domain gap for certain intensity and spatial features that are not encompassed within both the synthesis protocol and training cohort. For instance, a model optimized with synthetic data exclusively derived from scans of healthy adults may struggle to adapt to images featuring significant pathology or those from infants. In Section 5, we elaborate on our ongoing and future efforts to improve and expand our framework for enhanced model applicability for diverse imaging conditions and populations.

## 4 Current Applications

**Fig. 2.**
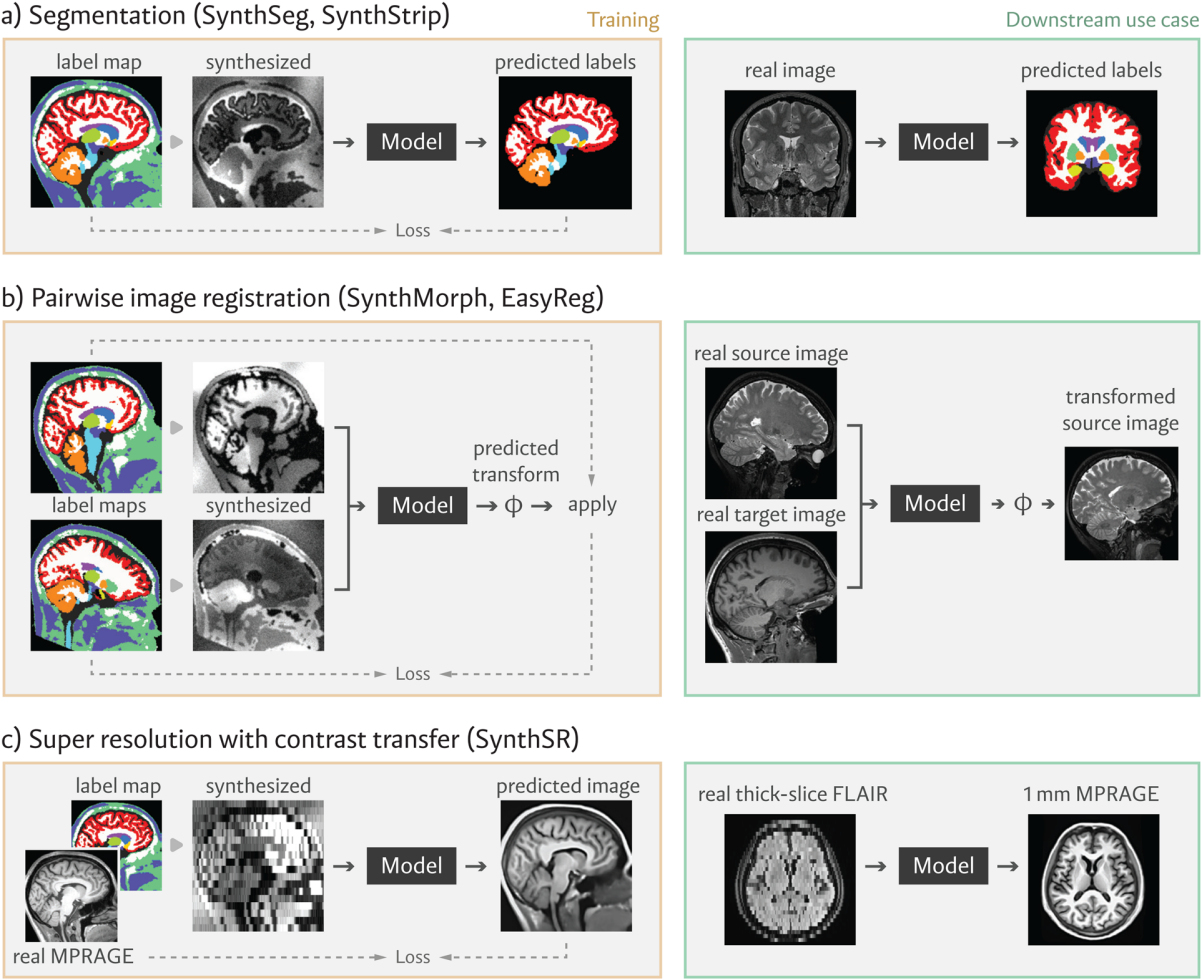
Applications using our synthesis framework. These include robust, contrast-agnostic methods for (a) whole-brain segmentation and skull-stripping, (b) linear and non-linear alignment, and (c) super-resolution and contrast transfer to MPRAGE characteristics. For each tool, orange boxes on the left illustrate the model optimization procedure using synthetic data, and green boxes on the right demonstrate downstream use of the model at inference time.

Over the past few years, our group has successfully applied this synthesis strategy to tackle several neuroimaging problems. This section reviews these deployed applications, illustrated in Figure 2.

### 4.1 Whole-brain segmentation and skull stripping

SynthSeg, the first tool to encapsulate our synthesis-based strategy, is a contrast-agnostic model that segments any brain MR scan into 32 anatomical regions (Billot, Greve, Leemput, et al., 2020; Billot, Greve, Puonti, et al., 2023; Billot, Robinson, et al., 2020). We optimize SynthSeg using synthetic images as input and components of the deformed source label map $s_{\phi }$ as the target output (Fig. 2a). By randomly downsampling and smoothing synthetic inputs, we train SynthSeg to produce 1 mm isotropic segmentations even for low-resolution images at inference; we note that, while this output resolution is constant, the predictions are expected to be crisper and more accurate for higher-resolution input scans. A subsequent iteration of SynthSeg (Billot, Magdamo, et al., 2023) includes four crucial improvements that enable neuroimaging studies of uncurated clinical MRI data directly downloaded from the picture archiving and communication system (PACS) of a hospital. First, a hierarchical approach greatly enhances the robustness of the method by segmenting four tissue types. We ensure the plausibility of the tissue segmentation with an autoencoder and segment the brain regions conditioned on the tissue types. Second, we subdivide of the cortex into parcels. Third, we estimate the intracranial volume, an important covariate in volumetric studies. Fourth, we include an automatic quality control module that automatically rejects poorly segmented scans.

SynthStrip is another contrast-agnostic segmentation model that we release (Hoopes, Mora, et al., 2022; Kelley et al., 2024). This tool provides a binary image delineation of brain and non-brain voxels—a task commonly known as skull stripping or brain extraction (Eskildsen et al., 2012; Iglesias et al., 2011; Ségonne et al., 2004; S. M. Smith, 2002). This is a fundamental first step in many neuroimaging pipelines as it removes irrelevant features from the image and facilitates downstream analyses like brain-specific registration. During training, SynthStrip learns to predict a signed distance map to the brain matter boundary, and unlike earlier deep learning-based skull stripping algorithms (Kleesiek et al., 2016; Roy et al., 2017; Salehi et al., 2017), we leverage the synthesis strategy to achieve robust brain extraction across a variety of data types.

### 4.2 Image registration

Medical image registration estimates a spatial transformation between corresponding anatomies of two images. Classical methods rely on iterative optimization approaches (Ashburner, 2007; Avants et al., 2008; Modat et al., 2010; Rueckert et al., 1999), whereas modern learning-based registration methods directly predict a deformation from two input scans using a neural network. A number of early learning registration approaches optimize a loss comparing the network output to a ground truth warp (Krebs et al., 2017; Rohé et al., 2017; Yang et al., 2017; Young et al., 2022). More recently, unsupervised methods train with image similarity functions similar to those of classical methods (Balakrishnan et al., 2019; De Vos et al., 2019; Hoopes, Hoffmann, et al., 2022; Krebs et al., 2019). While these techniques yield high accuracy in intra-modality registration, they inherit the limitations of classical techniques (lack of robustness) when registering across modalities (Iglesias et al., 2013).

In SynthMorph (Hoffmann, Billot, et al., 2021; Hoffmann et al., 2022, 2023, 2024) and its extension EasyReg (Iglesias, 2023), we train a registration network with synthetic images of random resolution and contrast, circumventing the need for inter-modality similarity measures. Specifically, these methods rely on generating two, rather than one, synthetic images from the same label map, using different random deformations, resolutions, and appearances (Fig. 2b). The network can then be trained to predict deformations in a supervised or unsupervised fashion. The former relies on the known ground truth deformation similar to Young et al. (2022), whereas the latter maximizes a measure of structure overlap, such as the Dice score, using the source label maps ϕ. EasyReg extends SynthMorph by estimating a deformation field in both registration directions. The forward deformation is averaged with the inverse of the reverse deformation to guarantee bi-directional symmetry.

We have also explored the possibility of training SynthMorph with label maps drawn from discrete, spatially smooth noise distributions, such as Perlin noise (Hoffmann et al., 2022). A network trained with such data enables registration of any anatomical region, at the expense of a slight loss of performance with respect to the dedicated brain model when registering brain MRI scans. Even for imaging modalities or body parts where the model may underperform, it is still useful as an unbiased starting point to initialize model training (Dey et al., 2022).

### 4.3 Super-resolution and contrast transfer

Synthetic images can also be used to solve other voxel-wise regression problems, such as super-resolution and contrast transfer. A super-resolution network (Iglesias et al., 2020, 2022) can be trained using low-resolution synthetic images as input and the corresponding high-resolution synthetic images as the targets. A similar strategy can be used to train networks for MRI contrast transfer (e.g., FLAIR to T1w), using synthetic images produced with our generator. SynthSR (Iglesias et al., 2023) achieves super-resolution and contrast transfer simultaneously (Fig. 2c), producing 1 mm isotropic T1w volumes from brain MRI scans of any slice direction, resolution, and contrast. This synthetic T1w is compatible with most existing morphometric tool for brain MRI analysis.

Training SynthSR requires real 1 mm T1w scans matching the label maps. In practice, this is not prohibitive since label maps are typically obtained from real T1w scans on healthy subjects in the first place. These T1w images are deformed by ϕ and used as regression targets by a neural network that seeks to predict them from synthetic input scans of random resolution and contrast. We optimize the network with a primary similarity loss using the sum of absolute image intensity differences between the prediction and the ground truth. When used in isolation, this loss yields predictions that are blurrier than ideal. To address this, we employ a second loss based on the Dice overlap between $s_{\phi }$ and the automated segmentation of the predicted images, estimated by a segmentation network. The weights of this second network are frozen during training, thus encouraging SynthSR to generate images that are well segmented and thus well defined at anatomical boundaries.

## 5 Ongoing and Future Work

In this section, we discuss ongoing and future efforts to apply our synthetic framework and various aims to enhance the effectiveness of synthesized images.

### 5.1 Improving synthesized images

Synthesizing image intensity features by recoding anatomical label maps may not fully capture the nuanced characteristics that exist in real acquisitions, as the anatomical detail of generated images are constrained by the granularity of the underlying segmentations. In some cases, this limitation might lead to trained models that do not fully match the within-domain performance of a model trained on a *specific* acquisition type. While one solution is to leverage high-resolution, densely-segmented maps with hundreds of anatomical regions, this is exceedingly difficult to acquire manually or even through automated means. A less prohibitive solution involves integrating real data and alternative synthesis methods alongside our framework to further expand the distribution of tissue characteristics seen by the model during training. This could involve occasionally applying different synthesis techniques (or none at all) or combining multiple synthesized images from various techniques into a single, joint image.

One useful alternative synthesis method capitalizes on MRI signal models to simulate *realistic* images with diverse acquisition characteristics via tissue parameter maps (Jacobs et al., 2024; Varadarajan et al., 2021). Specifically, pulse sequences like DESPOT2 (Deoni et al., 2005), MP2RAGE (Marques et al., 2010), and multi-echo GRE (S. Shin et al., 2023) compute magnetic property maps of the tissue, such as T1 and T2* relaxation time and proton density (PD). These maps can be combined as input to a forward model derived from the Bloch equation (Bloch, 1946; D. Ma et al., 2013) to generate images that closely mirror the appearance of a real acquisition. By randomly varying sequence parameters in the Bloch simulation, we can generate realistic images with a range of tissue contrasts, all from a single acquisition. However, with this technique, images are not computed directly from a label map and therefore require more precise image annotations to train accurate models in supervised scenarios.

Another approach to synthesis involves generative modeling techniques that learn to reconstruct new brain images from input label maps (Fernandez et al., 2022; Pinaya et al., 2022). Looking forward, multi-modal neuroimaging models could further augment our framework with fine-grained control, for instance by using a natural language prompt to specify desired image properties or alterations. While these models tend to interpolate between features seen in real data and do not substantially exceed characteristics of the training domain, they provide a way to generate detailed images from labels used to supplement our framework.

A related avenue of future research is conditional generation of synthetic scans (Chambon et al., 2022; Fernandez et al., 2022; Pinaya et al., 2022), given non-imaging variables such as age and gender. Modeling such variables may not be trivial (e.g., how does the “age” of a brain scan change if a random nonlinear augmentation expands the ventricles?), but would open the door to applications such as age prediction (“BrainAge”), which is a reliable biomarker for cognitive impairment and several diseases (Cole et al., 2017).

### 5.2 Modeling imaging artifacts

By simulating acquisition errors and distortions, we can potentially train models that learn to extract and correct these corruptions in real images. In MRI, the acquisition is performed in the frequency domain (*k*-space), and errors in the frequency domain can have a rather broad effect on the image, such as localized distortions or spurious sinusoidal patterns (Graves & Mitchell, 2013; Jezzard & Balaban, 1995). Modern MRI acquisitions also often undersample *k*-space through the use of parallel imaging or compressed-sensing (Griswold et al., 2002; Lustig et al., 2008; Pruessmann et al., 1999), which require more advanced reconstruction algorithms that cause specific artifacts (Brau et al., 2008). The use of slice-selective pulses can also lead to spin-history artifacts, caused by imperfect pulse shapes (Malik et al., 2011) or head motion (Friston et al., 1996)—which itself can also disrupt the consistency of k-space and lead to smearing and duplicated edges (Zaitsev et al., 2015). Each of these phenomena can be synthesized in brain images, either using rough approximations or by simulated physical processes with supplemental data.

Synthetic images also have use for bias field estimation and correction of both B1- (receive) and B1+ (transmit) inhomogeneities. The receive field is an intensity scaling across space and easily simulated in its general appearance as a spatial distribution of high sensitivity regions near coils with a rapid fall-off depending on the size of the coil (so higher sensitivity scaling also results in more rapid fall-off from smaller coils). While this type of profile is simple to approximate in image-based augmentation, doing so yields an image with a bias field that is the composition of the true bias field and the simulated one.

In contrast, one can include the receive bias field directly into the image synthesis procedure so it is the only one present in the resulting data. On the transmit side, the steady state Bloch equations (Bloch, 1946) can be integrated into the synthesis, as long as parameter maps are available. In that case, the transmit bias field is applied to a randomly generated flip angle to yield a “flip angle field” that modulates the contrast of the image in a tissue-dependent fashion through the Bloch equations. A receive bias field can then be applied after the initial image formation from the magnetic properties.

While modeling the precise form of the B1+/- fields is difficult, simulating a wider range of variation than we expect to see in practice may be sufficient to learn to disentangle and separately predict the fields. Alternatively, one could also use an adversarial approach to simulate realistic bias fields that maximize the performance of a downstream tasks, for example, image segmentation (C. Chen et al., 2020).

### 5.3 Targeted anatomical analysis

Brain synthesis methods promise to improve existing anatomy-specific analysis approaches in various contexts.

#### 5.3.1 Surface reconstruction

Cortical reconstruction techniques seek to localize and model the tissue boundaries of the white matter and pial surfaces, to facilitate accurate analysis of the morphological, functional, and connective organization of the cortex. Current tools like FreeSurfer rely on time-consuming, per-image optimization methods (Dale et al., 1999; Fischl et al., 1999), but recent learning-based approaches offer solutions to efficient and accurate reconstruction (Bongratz et al., 2022; Cruz et al., 2021; Q. Ma et al., 2021, 2022). To facilitate surface fitting across a range of image contrasts, we are augmenting our cortical reconstruction tools (Gopinath et al., 2023, 2024; Hoopes, Iglesias, et al., 2022) by leveraging a mix of real and synthetic images during training. Our straightforward label-to-image synthesis does not reproduce the intensity features necessary for proper reconstruction of highly-folded cortical convolutions. To address this, we are currently leveraging more realistic synthesis methods based on parameter maps, as described in Section 5.1.

#### 5.3.2 Brain vasculature

The vascular anatomy is of great interest both for research and medical applications (stroke, atherosclerosis, aneurysm). Arteries are typically imaged using variants of MR angiography (contrast-based, time-of-flight), while veins are detected using susceptibility imaging (susceptibility-weighted imaging, quantitative T2*, quantitative susceptibility mapping). Finer levels of neurovasculature can be visualized in high-resolution *post mortem* MRI or using microscopy techniques.

Vascular modeling, therefore, requires the acquisition, segmentation, and analysis of multimodal, multiscale, cross-sectional data. Vessel segmentation has historically relied on Hessian filters that leverage the specifically anisotropic nature of image curvature around vessels (Frangi et al., 1998; Lorenz et al., 1997; Sato et al., 1998). However, these filters are local, sensitive to noise, and require tedious parameter tuning. Deep learning has shown promise, but vessel contrast varies greatly across scales and modalities, thereby requiring relabeling (which is particularly tedious and thus expensive) and retraining for each new case.

In this context, the synthesis of heterogeneous training data for vascular analysis is extremely appealing. There is a large body of literature concerned with the generation of realistic synthetic vascular networks, to accurately model the dynamics of blood flow and blood oxygenation. An early computation method is “constrained constructive optimization” (CCO), originally developed by Neumann et al. (1995) and later refined by their group and others (Karch et al., 1999, 2000; Schneider et al., 2012; Szczerba & Székely, 2005). Its use for training and validating segmentation algorithms was first proposed in Hamarneh and Jassi (2010) and was recently applied to the problem of retinal OCT segmentation (Menten et al., 2022). We believe that combining domain randomization techniques with vascular simulators has great potential in enabling vascular segmentation in a wide array of neuroimaging applications.

#### 5.3.3 Change detection and longitudinal analysis

Longitudinal within-subject image analysis approaches have the potential to increase the sensitivity and specificity of population studies. These methods can improve the efficiency of trials by requiring fewer subjects and providing surrogate endpoints to assess therapeutic efficacy. Current analysis tools effectively detect longitudinal changes in well-curated research data, such as ADNI. In these datasets, scan protocols are harmonized across acquisition sites to minimize differential distortions, and residual distortions, such as gradient nonlinearities, are corrected before data release.

Unfortunately, these tools fail in the presence of acquisition differences between scans. Such differences are ubiquitous in imaging collections, and especially prevalent in clinical imaging, where scheduling a subject on the same scanner and scan protocol as a previous session is difficult or impossible. The tools perform poorly in many challenging scenarios where they are faced with complex noise, anatomical atrophy, and varying MRI contrast and distortion across serial scans. It is, therefore, important to develop tools that can ignore large-scale technology-induced differences in the longitudinal scans, while finding subtle anatomical changes that indicate early disease processes such as atrophy in Alzheimer’s Disease (AD).

To overcome these barriers, the synthesis strategy presents an opportunity to detect potentially subtle neuroanatomical change, in the presence of large image differences coming from (uninteresting) aspects of the acquisition process, including field strength, receive coil, sequence parameters, gradient nonlinearities, and B0-distortions/read-out directions. Such artifacts make it challenging to obtain ground truth in real datasets. We believe that the synthesis strategy can be expanded to the longitudinal case, where both the target neuroanatomical changes and the nuisance effects can be modeled during image generation, and hence separately predicted during network training (Bernal et al., 2021; Karaçali & Davatzikos, 2006; Khanal et al., 2017; Larson & Oguz, 2022; Rusak et al., 2022). This strategy promises to enable longitudinal analysis and change detection from large-scale, potentially clinical-quality, image collections that was not previously possible.

#### 5.3.4 Brain development and myelination

The morphology of the brain quickly changes during childhood, followed by a long period of very slow changes during adulthood (Bethlehem et al., 2021). However, medical image processing pipelines are set to work on normative adult brains and overlook challenges present in early and late life. During childhood, maturation of different brain regions takes place at different periods and rates, yielding large differences in structure size, MRI signal, and image contrast between development stages and compared to adult brains (Dubois et al., 2021).

One example of such change is myelination, the complex process of insulating neuronal axons, which enables rapid communication across the brain. Myelination varies across developmental stages (Monje, 2018), brain regions (Nieuwenhuys & Broere, 2017), and individuals (Van Essen & Glasser, 2014), and is also affected by various neurological disorders (Fields, 2008). Brain MRI analysis of myelinating brains in prenatal and infant MRI is particularly challenging, for two reasons: the progressive flip in image contrast between gray and white matter, and the multi-modal distribution of white matter intensities due to varying levels of myelination. Synthetic training data offer an attractive solution to this problems (Shang et al., 2022): the contrast flip is modeled by the random distribution of Gaussian means, whereas the spatially varying myelination of the white matter can be modeled by subdividing this tissue type into sub-labels with different myelin levels—which can be done automatically by applying clustering algorithms to the intensities of the training images.

#### 5.3.5 Ex vivo MRI

The analysis of *ex vivo* imaging data (Augustinack et al., 2005; Edlow et al., 2019) presents unique challenges due to variability in anatomical structures and appearances from changes in the packing container and liquid. For example, the image may contain one hemisphere or both hemispheres, either with or without a brainstem or cerebellum. Cerebral vessels can contain residual iron content in some locations and not others, varying the contrast properties of the vasculature. Proton-free fluids such as fomblin can be used for packing so that the exterior is dark, or water-based fluids can be used, resulting in a bright exterior in many MRI contrasts. These effects are difficult to model in standard image-based augmentation. Nevertheless, in our synthesis framework, neural networks can be trained to be robust to the absence of structures by probabilistically removing or retaining during training.

### 5.4 Synthesis of brain abnormalities

Inpainting methods are used to remove pathology from disease-case data, such as white matter lesions or brain tumors (Iglesias et al., 2023; Prastawa et al., 2009; H. Zhang et al., 2020), or to inject synthetic lesions into scans of healthy subjects (da S Senra Filho et al., 2019). The former is advantageous for generating synthetic, “healthy” data that can be analyzed with image processing methods that may fail in the presence of severe abnormalities; the latter can be used to generate large amounts of data with known ground truth.

In the context of our synthesis framework, we have shown that our random Gaussian model combined with existing segmentations is sufficient to capture the appearance of white matter hyper- or hypo-intensities due to multiple sclerosis lesions (Billot et al., 2021). Integrating other types of abnormality with improved models of appearance has the potential to enable the automated analysis of large amounts of heterogeneous data from PACS around the world, with minimal or no curation. While modeling the appearance of common pathologies like stroke or tumors is certainly more difficult than for white matter hyperintensities, the availability of large public datasets with manual delineations (Liew et al., 2017; Menze et al., 2015) should facilitate this endeavor. For more uncommon pathologies, the absence of manual labels is the main obstacle to training machine-learning models. An appealing solution consists of generating such labels from scratch, using random shapes (Hoffmann et al., 2022), geometric rules (Dey et al., 2024), or more advanced constructive synthesis (Georg et al., 2010; Menten et al., 2022; Schneider et al., 2012), with the hope that the synthesized data make the network robust against the presence of pathologies that are not present in the training dataset.

### 5.5 Applications in neuropathology

Synthesizing training data has potential applications for neuroimaging to neuropathology correlation. These studies aim to correlate gold standard histopathological diagnoses and microscopic measurements with macroscopic biomarkers and morphometry (Charidimou et al., 2020; Edlow et al., 2018; Nolan et al., 2021; Webster et al., 2021). In the absence of timely ante mortem MRI or cadaveric imaging, such studies rely on *ex vivo* MRI scans for which training data are scarce. As explained above, our synthesis framework allows the construction of *ex vivo* analysis tools that benefit from the wealth of data available for *in vivo* MRI.

In a similar vein, such training data can be applied to new volumetric imaging modalities. For example, one proposed solution to the difficulty of image acquisition for neuroimaging to neuropathology correlation is the construction of 3D volumes from abundant 2D dissection photography (Gazula et al., 2023; Tregidgo et al., 2020). Modifying resampling and bias augmentations to account for 2D slices during training allows the generalization of existing domain randomization tools to these images, without the need for a large training set from an entirely new modality.

### 5.6 Universal models within and beyond neuroimaging

In most existing frameworks we discussed, synthesis is used to build robust models tackling a *single* task, such as registration or segmentation of specific regions of interest. More recently, more *general* neuroimaging models also employ these synthesis strategies but aim to tackle multiple tasks at once. For example, Neuralizer is an in-context learning framework that can solve new neuroimaging tasks at inference (Czolbe & Dalca, 2023). As input, it takes an image to be processed along with pairs of input-output examples that illustrate the task to be executed. Neuralizer employs synthetic images derived with our framework to ensure robustness to new tasks as well as new input and output image modalities and qualities. Additionally, synthesis can be used to train robust, multi-task models that learn to extract broadly useful neuroimaging features, functioning as initial training checkpoints for rapid fine-tuning of single-task objectives (Chua & Dalca, 2023; P. Liu et al., 2023). Finally, these approaches can be expanded beyond brain imaging, for example, by using label maps of random shapes to train generalized medical imaging tools. Existing methods achieve anatomy-invariant solutions for registration (Hoffmann et al., 2022, 2024), in-context segmentation (Butoi et al., 2023), interactive segmentation (Wong et al., 2023), and star-convex instance segmentation (Dey et al., 2024).

## 6 The Curse of Dimensionality and Modeling Limitations

Brain MRI scans that are acquired in clinical routine imaging are not only variable in terms of intensity contrast and resolution properties—which many of our synthesis-based tools successfully address—but also along several other dimensions: number and type of contrasts that are acquired, administration of a contrast agent, brain disorders not seen during training (as explained in Section 5.4), or the number of timepoints (and time interval between scans) in longitudinal imaging.

While data synthesis can conceivably cover each of these dimensions of variation when taken individually, training models that address all of them *simultaneously* incur the “curse of dimensionality,” the amount of data that needs to be synthesized grows *exponentially* with the number of dimensions of variation that the models should be robust against.

Furthermore, differences in acquisition and reconstruction strategies (e.g., compressed sensing or parallel imaging) can yield correlations across spatial locations and spatially varying noise distributions (Breuer et al., 2009; Griswold et al., 2002; Lustig et al., 2008; Pruessmann et al., 1999), which are currently not captured by our simplified model. In a similar manner, advanced machine-learning MRI reconstruction methods (e.g., AUTOMAP; Zhu et al., 2018) generate highly structured and signal-dependent noise that cannot be modeled analytically. These differences in noise distribution may disturb neural networks, especially when the signal-to-noise ratio is low, as in low-field MRI. More importantly, in the context of quantitative MRI (relaxometry, diffusion, perfusion), quantitative estimates can become heavily biased if the correct distribution is not taken into account, either during data sampling or in the loss calculation (Sijbers et al., 1999; Varadarajan & Haldar, 2015).

Tackling these problems will likely require training with synthetic data generated with advanced simulations (as discussed in Section 5.1 above), which will most probably be too computationally expensive to run on the fly on a common server. Circumventing this limitation may require supercomputing power, offline generation of a large (but finite) number of samples, or the development of computationally efficient approximations that yield comparable performance.

## 7 Discussion and Conclusion

In this article, we have reviewed our recently proposed paradigm to train neural networks with synthetic data, along with several applications that benefit (or have the potential to benefit) from the framework.

One area of potential impact is clinical neurology and translational neuroscience (Goetz, 2007; Matthews et al., 2006; Simon et al., 2009), via analysis of clinical *in vivo* MRI scans (C. Zhao et al., 2020). For the broad spectrum of neurological disorders associated with focal brain lesions, such as traumatic brain injury, ischemic stroke, intracerebral hemorrhage, or encephalitis, our understanding of the anatomic correlates of cognitive, physical, and behavioral symptoms has been hindered by an inability to process anisotropic images or lesioned brains in neuroimaging software platforms (Iglesias et al., 2023). Lesions often compromise the accuracy of cortical surfaces that are essential to generate volumetric measurements, leading to the exclusion of lesioned brains from clinico-radiologic correlation studies (Nuesch et al., 2009). Fundamental problems in neuroscience with profound implications for clinical neurology, such as understanding neural correlates of consciousness, remain unanswered, partly because precise MRI mapping of lesions is often infeasible in patients with disorders of consciousness (Edlow et al., 2024). By making it possible to generate cortical surfaces in images of lesioned brains acquired with arbitrary resolution and contrast, our techniques can create new opportunities to map lesions onto canonical brain atlases in 1 mm stereotactic space—thereby identifying the precise anatomic correlates of a broad spectrum of neuropsychological symptoms (Johnson et al., 2012; Montoya et al., 2006; Politis, 2014).

Our tools also have the potential to create opportunities to perform retrospective studies that mine massive clinical datasets whose potential has not been tapped. Clinical MRI scans that were previously only amenable to qualitative analysis can now be tested for associations between cortical and subcortical volumetric measures and clinical syndromes. Moreover, a large number of patients with neurodegenerative disorders worldwide have had serial clinical MRI scans during decades of care (Rinck, 2019). The integration of our tools with longitudinal analysis pipelines may generate insights into patterns of brain atrophy that have not been possible in prospective studies. In short, our tools can create opportunities to retrospectively study clinical MRI datasets, regardless of contrast, spatial resolution, or lesion burden, with sample sizes that are orders of magnitude larger than those of current prospective studies.

Finally, these strategies can impact domains where obtaining accurately labeled data is infeasible, such as longitudinal studies of cortical atrophy, or vessel segmentation (Chollet et al., 2024; Larson & Oguz, 2022; Rusak et al., 2022). These data hold promise in enabling supervised training of neural networks that achieve superhuman accuracy in these tasks (Azizi et al., 2023; Nikolenko, 2021). As the quality of simulations, the availability of data, and computational capabilities grow, learning-based methods relying on synthetic imaging data promise to play an important role in robust neuroimage processing tools.

## Data Availability

There are no data or code associated with this manuscript.
